# Yes1 signaling mediates the resistance to Trastuzumab/Lap atinib in breast cancer

**DOI:** 10.1371/journal.pone.0171356

**Published:** 2017-02-03

**Authors:** Tatsuaki Takeda, Hiromasa Yamamoto, Hirotaka Kanzaki, Ken Suzawa, Takahiro Yoshioka, Shuta Tomida, Xiaojiang Cui, Ramachandran Murali, Kei Namba, Hiroki Sato, Hidejiro Torigoe, Mototsugu Watanabe, Kazuhiko Shien, Junichi Soh, Hiroaki Asano, Kazunori Tsukuda, Yoshihisa Kitamura, Shinichiro Miyoshi, Toshiaki Sendo, Shinichi Toyooka

**Affiliations:** 1 Department of Clinical Pharmacy, Okayama University Graduate School of Medicine, Dentistry and Pharmaceutical Sciences, Okayama, Okayama, Japan; 2 Department of Thoracic, Breast and Endocrinological Surgery, Okayama University Graduate School of Medicine, Dentistry and Pharmaceutical Sciences, Okayama, Okayama, Japan; 3 Department of Clinical Genomic Medicine, Okayama University Graduate School of Medicine, Dentistry and Pharmaceutical Sciences, Okayama, Okayama, Japan; 4 Department of Biobank, Okayama University Graduate School of Medicine, Dentistry and Pharmaceutical Sciences, Okayama, Okayama, Japan; 5 Department of Surgery, Cedars-Sinai Medical Center, Los Angeles, California, United States of America; 6 Department of Biomedical Sciences, Cedars-Sinai Medical Center, Los Angeles, California, United States of America; University of South Alabama, UNITED STATES

## Abstract

**Background:**

Overexpression of human epidermal growth factor receptor 2 (HER2) is observed in approximately 15–23% of breast cancers and these cancers are classified as HER2-positive breast cancer. Trastuzumab is the first-line targeted therapeutic drug for HER2-positive breast cancer and has improved patient overall survival. However, acquired resistance to trastuzumab is still a critical issue in breast cancer treatment. We previously established a trastuzumab-resistant breast cancer cell line (named as BT-474-R) from a trastuzumab-sensitive *HER2*-amplified cell line BT-474. Lapatinib is also a molecular-targeted drug for HER2-positive breast cancer, which acquired the resistance to trastuzumab. Acquired resistance to lapatinib is also an issue to be conquered.

**Methods:**

We established trastuzumab/lapatinib-dual resistant cell line (named as BT-474-RL2) by additionally treating BT-474-R with lapatinib. We analyzed the mechanisms of resistance to trastuzumab and lapatinib. Besides, we analyzed the effect of the detected resistance mechanism in HER2-positive breast cancer patients.

**Results:**

Proto-oncogene tyrosine-protein kinase *Yes1*, which is one of the Src family members, was amplified, overexpressed and activated in BT-474-R and BT-474-RL2. Silencing of *Yes1* by siRNA induced both BT-474-R and BT-474-RL2 to restore the sensitivity to trastuzumab and lapatinib. Pharmaceutical inhibition of Yes1 by the Src inhibitor dasatinib was also effective to restore the sensitivity to trastuzumab and lapatinib in the two resistant cell lines. Combination treatment with dasatinib and trastuzumab induced down-regulation of signaling molecules such as HER2 and Akt. Moreover, the combination treatments induced G1-phase cell-cycle arrest and apoptosis. Consistent with cell line data, high expression of *Yes1* mRNA was correlated with worse prognosis in patients with HER2-positive breast cancer.

**Conclusion:**

*Yes1* plays an important role in acquired resistance to trastuzumab and lapatinib in HER2-positive breast cancer. Our data suggest that pharmacological inhibition of Yes1 may be an effective strategy to overcome resistance to trastuzumab and lapatinib.

## Introduction

Breast cancer is one of the most frequent malignancies for women in many countries [1–3]. Breast cancers are heterogeneous diseases, and are classified by the molecular characteristics. There are 5 intrinsic breast cancer subtypes: luminal A, luminal B, normal breast-like, human epidermal growth factor receptor 2 (HER2)-enriched, and basal-like, each unique in incidence, survival and response to the therapy [4].

HER2 is one of the receptor tyrosine kinases (RTK) comprising the HER protein family, and is located in the cell membrane [5]. HER family proteins, epidermal growth factor receptor (EGFR, HER1), HER3 and HER4 form heterodimers or homodimers following the activation by their ligands, and consequently, their dimerization leads to the activation of the downstream signaling such as the PI3K-Akt and MAPK signaling pathways, resulting in cell proliferation and survival [6]. The HER2-associated dimers generate the most powerful signaling for cellular transformation [7]. However, HER2 is known to have no ligands and forms the dimers in ligand-independent manner [7]. Thus, overexpression of HER2 is considered to be the oncogenic-driver in several malignancies including breast, gastric, esophageal and ovarian cancers [8, 9].

Trastuzumab is a first-line therapeutic drug for HER2-positive breast cancer, and the addition of trastuzumab to chemotherapy was associated with a higher rate of objective response, a longer duration of response and survival [10]. However, the acquired resistance to trastuzumab is known to be observed in most of the treated cases within one year after the treatment starts [11] and overcoming the acquired resistance to trastuzumab is one of the major clinical issues contributing to mortality of women. To date, various mechanisms of the acquired resistance to trastuzumab are reported such as the activation of other RTKs including EGFR, HER3, HER4 and MET [12, 13], the activation of the downstream signaling of HER2 [12, 13], T798M mutation in *HER2* gene [14], *PIK3CA* mutations [15], the activation of Src [16, 17], the expression of p95HER2 [12, 18], the elevated expression of Hsp90 [19], reduced expression of PTEN [15, 20], the expression of MUC4 [12, 13, 21], and the inhibition of miR200c [22].

To understand the molecular basis for resistance to HER2-targeted therapies, we previously established a trastuzumab-resistant breast cancer cell line (named as BT-474-R) from a trastuzumab-sensitive cell line with *HER2* amplification (BT-474) by treating BT-474 cells with increasing doses of trastuzumab [23]. Analysis showed that nuclear factor-kappa B (NF-kappaB) was constitutively activated in the BT-474-R cells mimicking the phenotype of basal-like breast cancers. Pharmacologic inhibition of NF-kappaB improved sensitivity of BT-474-R cells to trastuzumab. However, inhibition of NF-kappaB with trastuzumab alone was inadequate to sensitize BT-474-R cells, indicating that there may be other unidentified mechanisms mediating the acquired resistance to trastuzumab.

Lapatinib is the first dual kinase inhibitor of EGFR and HER2 tyrosine kinases [24]. This drug is used to treat HER2-positive breast cancer with acquired resistance to trastuzumab [25]. Acquired resistance to lapatinib is also an emerging issue in clinical management of breast cancer patients. As lapatinib is administered to HER2-positive breast cancers with acquired resistance to trastuzumab in clinical setting, it is valuable to establish a trastuzumab/lapatinib-dual resistant cell line from a trastuzumab-resistant cell line.

In the current study, we established the trastuzumab/lapatinib-dual resistant cell line (named as BT-474RL2) from BT-474-R, in order to elucidate the mechanism of the acquired resistance to trastuzumab and lapatinib, and to overcome the resistance.

## Materials and methods

### Ethics statement

This study was carried out in strict accordance with the recommendations in the Guide for the Care and Use of Laboratory Animals of the National Institutes of Health. The protocol was approved by the Animal Care and Use Committee of Okayama University (Permit Number: OKU-2014635). All surgery was performed under ketamine and xylazine anesthesia, and all efforts were made to minimize suffering. Detailed method for the animal experiments is described in S1 Method.

### Cell lines and reagents

HER2-positive human breast cancer cell line BT-474 (catalog number: HTB-20) was purchased from American Type Culture Collection (Manassas, VA, USA). BT-474-R was previously established by treating BT-474 with increasing doses of trastuzumab (from 0.1 μg/mL to 40 μg/mL) for 10 months [23]. To obtain trastuzumab/lapatinib-dual resistant BT-474 (named as BT-474-RL2), BT-474-R was additionally treated with increasing doses of lapatinib (from 0.1 μM to 5 μM) for 6 months. The cells were maintained in Dulbecco’s modified Eagle medium (DMEM) with 10% fetal bovine serum at 5% CO_2_ under 37°C. Trastuzumab was purchased from Chugai Pharmaceutical Co., Ltd. (Tokyo, Japan). Lapatinib was purchased from Cayman Chemical Company (Ann Arbor, MI, USA) and dasatinib was purchased from Bristol-Myers Squibb Company (New York, NY, USA).

### Cell viability assay

Cell proliferation was determined by CellTiter 96^®^ AQueous One Solution Cell Proliferation Assay (Promega, Fitchburg, WI, USA). Cells (3,000/well) were seeded in 96-well plates and then the medium in the wells was replaced with the medium containing diluted drug solutions of trastuzumab, lapatinib, dasatinib or their combinations in a 4-log range or complete medium, which were distributed in 8-replicate wells. Cells were incubated in the presence of each concentration of the respective drugs for 72 hours at 37°C in a humidified atmosphere of 5% CO_2_ in air. After that, MTS dye was added to each well. The cultures were incubated for another 1 hour at 37°C in a humidified atmosphere with 5% CO_2_. Optical densities of samples were measured at 492 nm using Multiskan^™^ FC Microplate Photometer (Thermo Fisher Scientific, Waltham, MA, USA). Mean optical density at each drug concentration was calculated after discarding the highest and lowest values. The anti-tumor effects of respective drugs for each cell line were shown in terms of inhibitory concentration at 25% (IC_25_) for trastuzumab or 50% (IC_50_) lapatinib, which was determined by plotting the graph of percentage of cell growth inhibition (Y-axis) versus drug concentration (X-axis). IC_25_ and IC_50_ values were expressed as mean and standard errors (SE). The assays were repeated more than three times.

### HER2 expression in BT-474 and its sublines

The expression of HER2 was determined by flow cytometry. The cells were harvested, washed and fixed by 4% formaldehyde for 10 min at 37°C. In the primary reaction, the cells were treated with either no antibody or anti-HER2 antibody (Cell Signaling Technology, Beverly, MA, USA) for 1 hour at 25°C. After washing, the cells were treated with Alexa Fluor ^®^-488-conjugated secondary antibody (Cell Signaling Technology) for 30 min at 25°C. After washing, the cells were analyzed using BD FACSCalibur (BD Biosciences, Franklin Lakes, NJ, USA). The cell population was gated on forward scatter and side scatter. The HER2 expression analysis was performed with CellQuest version 3.1 (BD Biosciences).

### Western blotting

Cells were washed in ice-cold PBS and lysed in RIPA buffer (Sigma-Aldrich, St. Louis, MO, USA). Cell lysate was collected after centrifugation at 15,000 rpm for 15 min at 4°C. Protein was quantitated using DC^™^ protein assay (Bio-Rad Laboratories, Hercules, CA, USA), fractionated on SDS-PAGE and blotted onto a membrane using Trans-Blot^®^ Turbo^™^ Transfer System (Bio-Rad Laboratories). After the membrane was blocked with 5% skim milk in TBS-containing 0.05% Tween 20 (T-TBS) for 1 hour, it was probed with various the primary antibodies overnight at 4°C, followed by the incubation with the secondary antibodies for 1 hour at 25°C. Proteins were detected using ECL Prime Western Blotting Detection Reagent (General Electric Company, Fairfield, CT, USA) and by scanning the membrane using ImageQuant LAS 4000 (General Electric Company). The antibodies used were as follows: phospho-EGFR-Tyr1068 (rabbit monoclonal antibody, catalog number: 2234S, dilution, 1:1000, Cell Signaling Technology), EGFR (rabbit monoclonal antibody, catalog number: 2085S, dilution, 1:1000, Cell Signaling Technology), phospho-HER2-Tyr877 (rabbit monoclonal antibody, catalog number: 2241S, dilution, 1:1000, Cell Signaling Technology), HER2 (rabbit monoclonal antibody, catalog number: 2165S, dilution, 1:1000, Cell Signaling Technology), phospho-HER3-Tyr1289 (rabbit monoclonal antibody, catalog number: 4791S, dilution, 1:1000, Cell Signaling Technology), HER3 (rabbit monoclonal antibody, catalog number: 4754, dilution, 1:1000, Cell Signaling Technology), phospho-Akt-Ser473 (rabbit monoclonal antibody, catalog number: 4060S, dilution, 1:1000, Cell Signaling Technology), Akt (rabbit monoclonal antibody, catalog number: 9272S, dilution, 1:1000, Cell Signaling Technology), phospho-MAPK-Tyr202/204 (rabbit monoclonal antibody, catalog number: 9101, dilution, 1:1000, Cell Signaling Technology), MAPK (rabbit monoclonal antibody, catalog number: 9102, dilution, 1:1000, Cell Signaling Technology), phospho-Src family-Tyr416 (rabbit monoclonal antibody, catalog number: 2101, dilution, 1:1000, Cell Signaling Technology), Src (rabbit monoclonal antibody, catalog number: 2109, dilution, 1:1000, Cell Signaling Technology), Yes1 (rabbit monoclonal antibody, catalog number: 3201S, dilution, 1:1000, Cell Signaling Technology), Actin (mouse monoclonal antibody, catalog number: MAB1501R, dilution, 1:5000, Merck Millipore, Darmstadt, Germany), anti-rabbit IgG-HPR (goat monoclonal antibody, catalog number: sc-2030, dilution, 1:2000, Santa Cruz Biotechnology, Santa Cruz, CA, USA) and anti-mouse IgG (goat monoclonal antibody, catalog number: sc-2031, dilution, 1:2000, Santa Cruz Biotechnology).

### Gene expression assays by quantitative reverse transcription PCR

Total RNA was isolated using RNeasy mini Kit (Qiagen, Venlo, Netherlands) and reverse transcribed with High Capacity cDNA Reverse Transcription Kit (Thermo Fisher Scientific, Waltham, MA, USA). The quantitative reverse transcription PCR (q-RT-PCR) was performed on StepOnePlus^™^ Real-Time PCR System (Thermo Fisher Scientific) using Power SYBR^®^ Green PCR Master Mix (Thermo Fisher Scientific). The gene expression was calculated using delta-delta CT method. Samples were analyzed in triplicate. *GAPDH* was used as an endogenous control. Data were expressed as mean and SE. The assays were repeated three times. The primers used were as follows. *GAPDH*: (F) 5’-TCG GAG TCA ACG GAT TTG GTC-3’, (R) 5’-AAA CCA TGT AGT TGA GGT CAA TG-3’, *c-Src*: (F) 5’-TGA AGA CAA TGA GTA CAC GGC-3’, (R) 5’-CTT TGT GGT GAG CTC AGT CA-3’, *Yes1*: (F) 5’-GAG AAT CTT TGC GAC TAG AGG-3’, (R) 5’-CTG GCA TCA TTG TAC CTG G-3’, *Fyn*: (F) 5’-CAC CGT CTT TGG AGG TGT G-3’, (R) 5’-TCA TCT TCT GTC CGT GCT TC-3’, *Fgr*: (F) 5’-AAC CCT GGC TTC CTT GAT AG-3’, (R) 5’-TTG GTG AAG GTG AGG TCA TC-3’, *Lyn*: (F) 5’-ATG TGA GAG ATC CAA CGT CC-3’, (R) 5’-TGC CAT CAT AGG GGT ACA AG-3’, *Lck*: (F) 5’-TCC CAT AGT CCC ACT GGA TG-3’, (R) 5’-CTG TGC AGA GCG ATA ACC AG-3’, *Hck*: (F) 5’-GGC CTA ATA GCC ACA ACA GC-3’, (R) 5’-TGA GGT CTT CGT GGT GAA TG-3’, *Blk*: (F) 5’-CTT CAA CCA CCT TAC TCC TCC-3’, (R) 5’-AGG TCC CGA TCA TTC ATA GC-3’, *Frk*: (F) 5’-CTG AGG ACA GAA GCC TAC AGG-3’, (R) 5’- GGA ACC GGT CTT GTT TTC TG-3’.

### Copy number assays by qPCR

DNA was isolated using DNeasy Blood & Tissue Kit (Qiagen). Copy number assays were determined by qPCR using Power SYBR^®^ Green PCR Master Mix (Thermo Fisher Scientific). The gene dosage was calculated using the standard curve method. Samples were analyzed in triplicate. *LINE-1* was used as a reference gene. Relative copy number of each sample was determined by comparing the ratio of target gene to *LINE-1* in each sample with the ratio of these genes in Human Genomic DNA (HGD) (Merck Millipore). Data were expressed as mean and SE. The assays were repeated three times. The primers used were as follows. *LINE-1*: (F) 5’-AAA GCC GCT CAA CTA CAT GG-3’, (R) 5’-TGC TTT GAA TGC GTC CCA GAG-3’, *Yes1*: (F) 5’-TTA CGG AAT CAT GCC ACT C-3’, (R) 5’-CCC ATG CCC AAT AAA GTG-3’.

### siRNA transfection

Small interfering RNAs (siRNAs) specific for *Yes1* (*Silence*r^®^ Select Validated siRNA #4390824) and non-targeting control (*Silence*r^®^ Select Negative Control No.2 siRNA #4390846) were purchased from Thermo Fisher Scientific. Each siRNA was transfected to the cells using Lipofectamine^®^ RNAiMAX Transfection Reagent (Thermo Fisher Scientific), incubated for 48 hours according to the manufacturer’s instructions.

### Clonogenic assay

Cells (1,000/well) were seeded in 6-well plates in triplicate. After the attachment to plastic surface, cells were treated with trastuzumab (1 μg/mL) alone, dasatinib (100 nM) alone or the combination of them for 14 days. After fixation by 4% formaldehyde, cells were stained using 0.2% crystal violet. Colony counts were measured using ImageJ version 1.48 (National Institutes of Health, Bethesda, MD).

Data were expressed as mean and SE. The assays were repeated three times.

### Soft agar colony formation assay

Cells (1,000/well) were suspended in 0.33% agar in DMEM with 10% fetal bovine serum and layered over a 0.50% agar base in 6-well plates in triplicate followed by the treatment with trastuzumab (1 μg/mL) alone, dasatinib (100 nM) alone or the combination of them for 24 hours. After 14 days, the number of microscopically visible colonies (>30 cells) was counted. Data were expressed as mean and SE. The assays were repeated three times.

### Cell-cycle analysis

Cells were seeded in 6-well plates. After attachment to plastic surface, cells were treated with trastuzumab (1 μg/mL) alone, dasatinib (100 nM) alone or their combination for 24 hours. The cell cycle distribution was assessed using a propidium iodide staining-based assay with the Cycletest^™^ Plus DNA Reagent Kit (BD Biosciences) and FACSCaliber (BD Biosciences). The cell cycle analysis was performed with CellQuest version 3.1 (BD Biosciences). Data were expressed as mean and SE. The assays were repeated three times.

### Apoptosis analysis

Cells were seeded in 6-well plates. After attachment to plastic surface, cells were treated with trastuzumab (1 μg/mL), dasatinib (100 nM) or their combination for 48 hours. And then cell lysate was collected for western blot analysis. The level of cleaved PARP was examined using the antibody for cleaved PARP (mouse monoclonal antibody, catalog number: 9546S, dilution, 1:2000, Cell Signaling Technology).

### Bioinformatic analysis

Messenger RNA profiling for 236 breast cancer patients (GSE3494) and 204 breast cancer patients with relapse (GSE12276) were obtained from GEO (http://www.ncbi.nlm.nih.gov/geo/). Briefly, patients were divided into low and high expression group of *HER2* or *Yes1* mRNA, based on the median expression value of all the patients within the dataset, respectively. Disease-specific survival (DSS) of the cases with higher expression of *Yes1* was compared to that of the cases with lower expression of *Yes1* for the patients with higher expression of *HER2* in GSE3494 dataset, while relapse-free survival (RFS) was compared using GSE12276 dataset with higher expression of *HER2*.

### Statistical analysis

All the statistical analyses in this study were performed using EZR version 1.32 (Saitama Medical Center, Jichi Medical University, Saitama, Japan), which is a graphical user interface for R version 3.2.2 (The R Foundation for Statistical Computing, Vienna, Austria) [26]. Specifically, the software is a modified version of R commander designed to add statistical functions frequently used in biostatistics. The difference was compared using one-way analysis of variance (multiple groups) or t-test (two groups). The disease-specific survival (DSS) or relapse-free survival (RFS) was calculated using two different cohorts. The survival curve was calculated by the Kaplan—Meier method and the difference between groups was compared with the log-rank test. A value of *P* < 0.05 was considered statistically significant.

## Results

### Characterization of trastuzumab-resistant cell line BT-474-R and trastuzumab/lapatinib-dual resistant cell line BT-474-RL2

**Fig 1:**
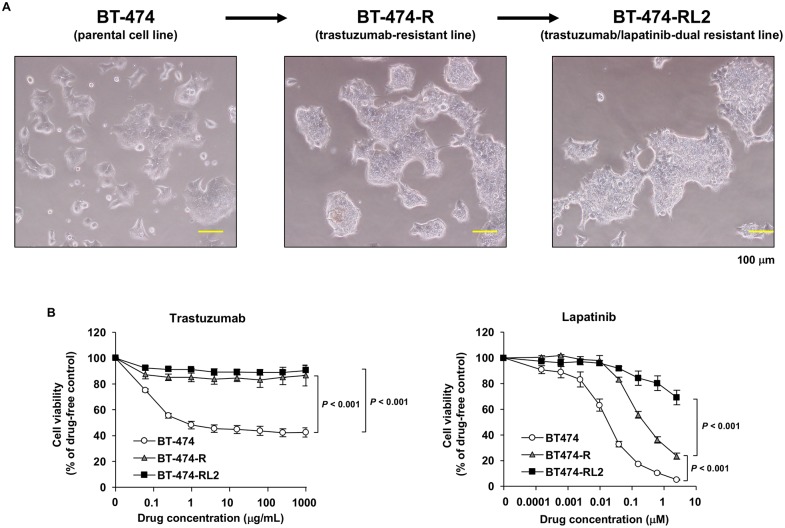
Establishment of trastuzumab-resistant cell line BT-474-R and trastuzumab/lapatinib-dual resistant cell line BT-474-RL2. (A) Morphological changes in parental cell line (BT-474) and its sublines (BT-474-R and BT-474-RL2). (B) MTS assay evaluating cell viability of BT-474, BT-474-R and BT-474-RL2 upon treatment with various concentrations of trastuzumab or lapatinib for 72 hours. Data are shown as means ± standard errors (SE). The assay using trastuzumab was repeated five times, and the assay using lapatinib was repeated three times.

Parental BT-474 cell line and established two cell lines BT-474-R and BT-474-RL2 were proven to have the same genetic origins and were authenticated using *GenePrint*^®^ 10 System (Promega). Morphological changes in the parental cell line and its sublines are shown in Fig 1A. To confirm the resistance to trastuzumab and lapatinib, we performed the cell viability assay (Fig 1B). The IC_25_ value of BT-474, BT-474-R and BT-474-RL2 for trastuzumab was 0.114 ± 0.0314, > 1000 and > 1000 μg/mL, respectively. As for lapatinib, the IC_50_ value of them was 0.0178 ± 0.00285, 0.239 ± 0.0443 and 3.94 ± 0.225 μM, respectively (Table 1).

**Table 1:** IC_25_ and IC_50_ values of BT-474 and its sublines.

Cell line	Characteristics	IC_25_ (mean ± SE)	IC_50_ (mean ± SE)
		Trastuzumab (μg/mL)	Lapatinib (μM)
BT-474	*HER2* amplification	0.114 ± 0.0314	0.0178 ± 0.00285
BT-474-R	Trastuzumab-resistant subline	> 1000	0.239 ± 0.0443
BT-474-RL2	Trastuzumab/lapatinib-resistant subline	> 1000	3.94 ± 0.225

**Fig 2:**
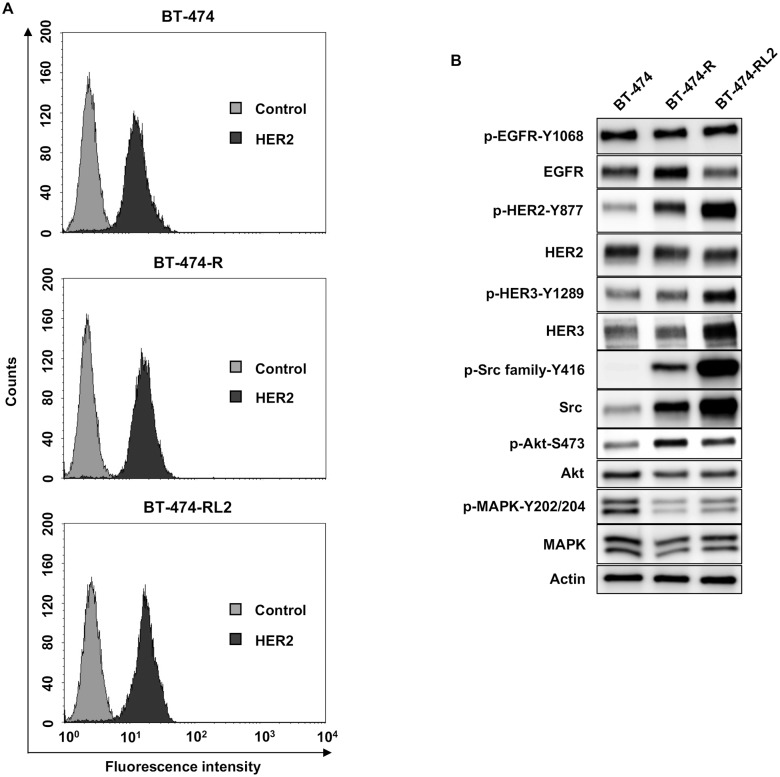
Expression of HER2 and its related proteins. (A) Flow cytometric analysis of HER2 expression level on BT-474, BT-474-R and BT-474-RL2. (B) Protein expression profile of BT-474, BT-474-R and BT-474-RL2. Phosphorylation of major cell signaling pathways changes among BT-474, BT-474-R and BT-474-RL2.

We next evaluated the expression of HER2 in cell surface among BT-474, BT-474-R and BT-474-RL2 by flow cytometry. There was no significant difference of the expression of HER2 among them (Fig 2A). We also elucidated the expression of HER2-related signaling. In resistant cell lines, the phosphorylation of HER2, Akt and Src family was upregulated. Phospho-MAPK was downregulated in resistant cell lines (Fig 2B).

### Yes1 is upregulated in BT474-R and BT474-LR resistant cell lines

**Fig 3:**
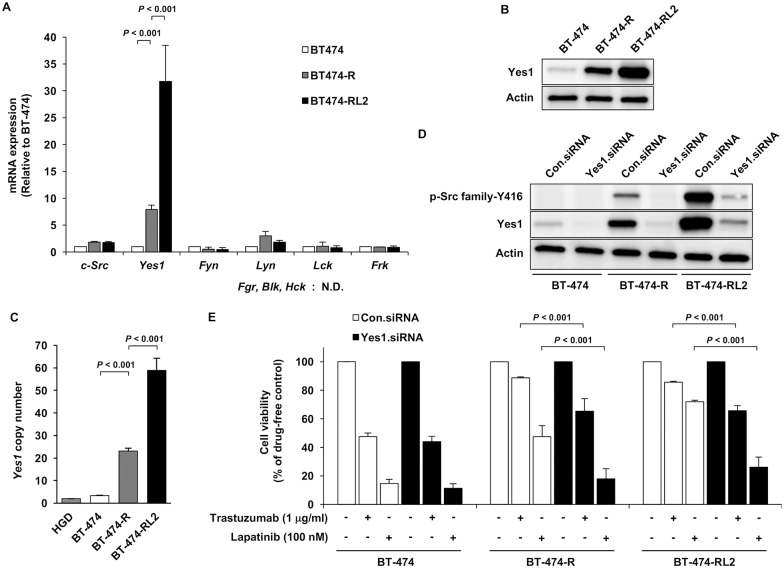
Yes1, one of the Src family member, is a key molecule in acquired HER2-resistance. (A) Comparison of mRNA expression of Src family members in BT-474, BT-474-R and BT-474-RL2 by q-RT-PCR. *Fgr*, *Blk* and *Hck* was not detected. Data are shown as means + SE. The assay was repeated three times. (B) Western blot analysis of Yes1 protein expression among BT-474, BT-474-R and BT-474-RL2. (C) Copy number assay of *Yes1* in BT-474, BT-474-R and BT-474-RL2. Human Genomic DNA (HGD) was used as the control (2 copies). Data are shown as means + SE. The assay was repeated three times. (D) Protein expression profile after Yes1 knockdown by *Yes1* siRNA in BT-474, BT-474-R and BT-474-RL2. (E) MTS assay assessing the sensitivity to trastuzumab or lapatinib among BT-474, BT-474-R and BT-474-RL2 after Yes1 knockdown. Cells (3,000/well) were seeded in 96-well plates and transfected with siRNA for 48 hours followed by the treatment of trastuzumab or lapatinib for 72 hours. Data are shown as means + SE. The assay was repeated three times.

Because expression of Src family protein was increased in the resistant cell lines, we evaluated mRNA expression of Src family proteins. Src family proteins consist of 9 members, that is, c-Src, Yes, Fyn, Lyn, Fgr, Blk, Hck, Lck and Frk. mRNA expression of *Yes1* was increased in the resistant cell lines, especially in BT-474-RL2, compared to other Src family proteins (Fig 3A). As for Fgr, Blk and Hck, the mRNA expression was not detected. To confirm the absence of mRNA expression of them, Burkitt’s lymphoma cell line Daudi (catalog number: JCRB9071) and histiocytic lymphoma cell line U937 (catalog number: JCRB9021) were used as positive controls for Fgr, Blk and Hck and conventional RT-PCR was performed. mRNA expression of Fgr, Blk and Hck was not detected in BT-474, BT-474-R or BT-474-RL2 (S1 Method, S1 Fig). Next, we performed western blot analysis to evaluate the protein expression of Yes1. Expression of Yes1 protein was considerably elevated (Fig 3B). We also determined the copy number of *Yes1*. Copy number of *Yes1* was gained in resistant cell lines (Fig 3C), which indicates the cause of the elevated expression of Yes1 was at partially due to its increased copy number.

### Effect of Yes1 in resistant cell lines

To clarify the effect of Yes1 in resistant cell lines, we performed siRNA-mediated knockdown of *Yes1*. After the administration of siRNA of *Yes1*, the expression of Yes1 was significantly inhibited. Predominant reduction of Yes1 protein resulted in the downregulation of phospho-Src family, suggesting that the activation of phospho-Src family was due to the upregulation of Yes1 (Fig 3D). We also confirmed that the effect of trastuzumab and lapatinib in the resistant cell lines were recovered after knockdown of Yes1 (Fig 3E).

### Effect of the Src inhibitor dasatinib in the resistant cell lines

**Fig 4:**
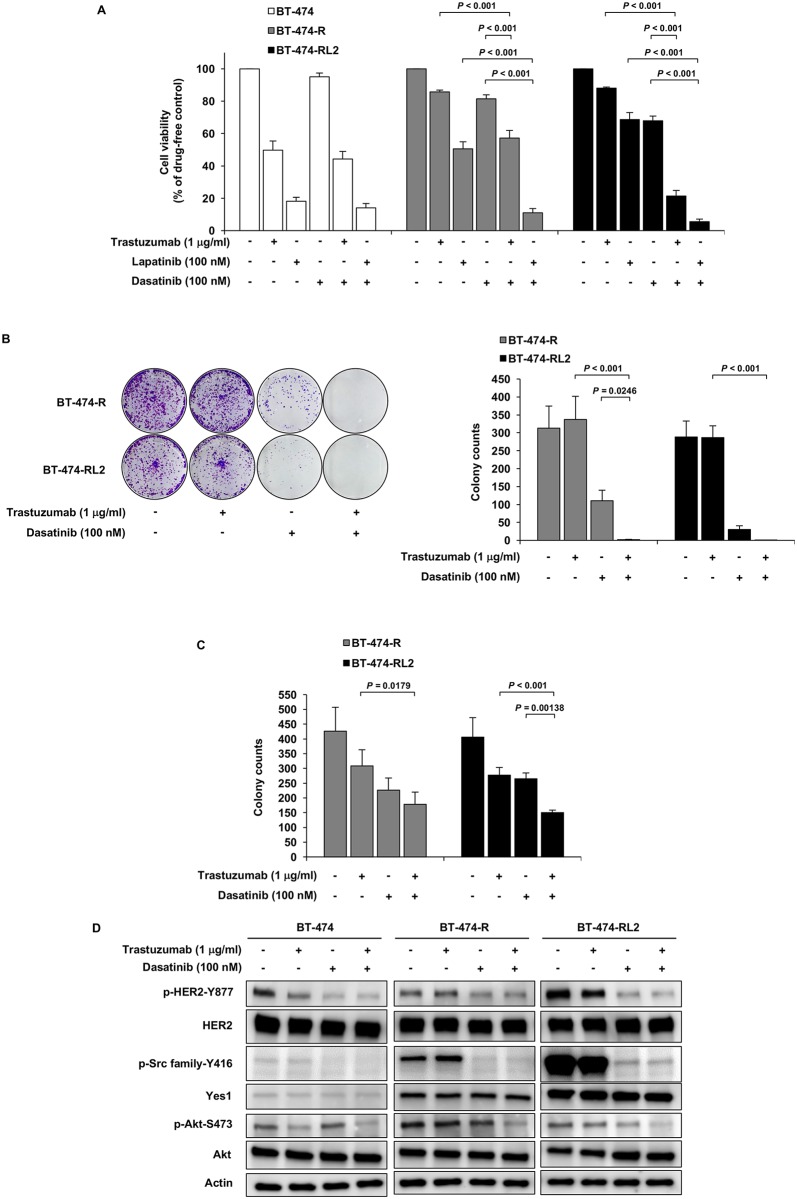
Dasatinib, Src family inhibitor, overcomes HER2-resistance. (A) MTS assay evaluating the effect of trastuzumab or lapatinib alone, dasatinib alone or the combination on BT-474, BT-474-R and BT-474-RL2. Data are shown as means + SE. The assay was repeated three times. (B) Clonogenic assay evaluating the effect of long-term exposure to trastuzumab alone, dasatinib alone or the combination of them on BT-474-R and BT-474-RL2. Data are shown as means + SE. The assay was repeated three times. (C) Soft agar colony formation assay in BT-474-R and BT-474-RL2. Data are shown as means + SE. The assay was repeated three times. (D) phosphorylation status of HER2 and Akt upon treatment with trastuzumab alone, dasatinib alone or the combination of them for 1 hour in BT-474, BT-474-R and BT-474-RL2.

Because Src family proteins, especially Yes1, was upregulated in the resistant cell lines, we focused on the Src inhibitor dasatinib. To overcome the resistance to trastuzumab, we performed the combination therapy of trastuzumab and dasatinib using cell viability assay and colony formation assays (clonogenic assay and soft agar colony formation assay). In the resistant cell lines, the combination of trastuzumab or lapatinib with dasatinib led to the additive anti-tumor effect (Fig 4A, 4B and 4C). We also performed western blot analysis to elucidate the effect of combination therapy to the HER2-related pathway. In BT-474-R and BT-474-RL2, phosphorylation of Akt was more inhibited in the combination therapy than in the single agent. Phosphorylation of HER2 was inhibited by dasatinib alone in all cell lines, which was similar to the previous report [16] (Fig 4D).

**Fig 5:**
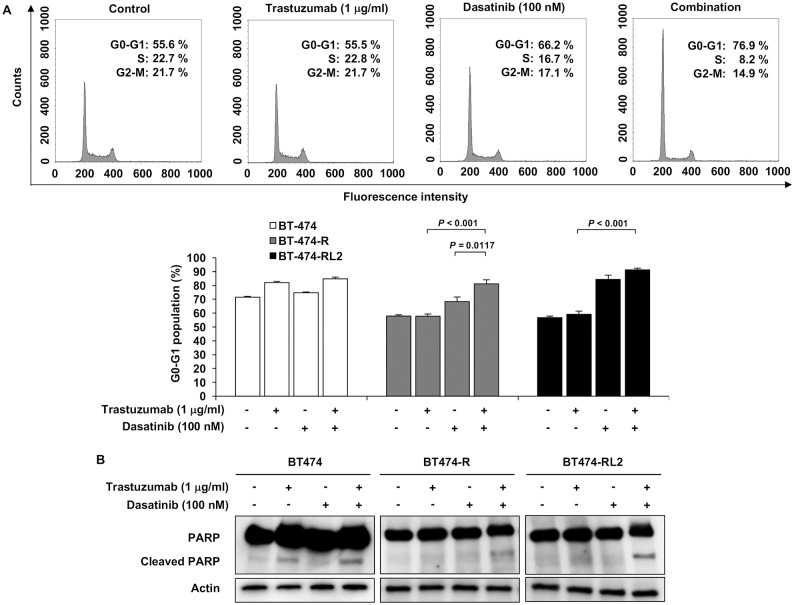
Combination of trastuzumab and dasatinib induce cell-cycle arrest in G1 phase and apoptosis. (A) Upper: Cell cycle analysis of BT-474-R upon treatment with trastuzumab alone, dasatinib alone or combination of them. Lower: G0-G1 population in BT-474, BT-474-R and BT-474-RL2. Data are shown as means + SE. The assay was repeated three times. (B) Apoptosis analysis by the detection of PARP cleavage in western blot analysis in BT-474, BT-474-R and BT-474-RL2.

Furthermore, we conducted cell-cycle analysis and apoptosis analysis to see the effect of trastuzumab and dasatinib in resistant cell lines. Combination therapy induced G1 arrest more (Fig 5A). Apoptosis was also induced after the combination of trastuzumab and dasatinib (Fig 5B).

### Prognostic significance of Yes1 mRNA expression in patients with higher expression of HER2 mRNA

**Fig 6:**
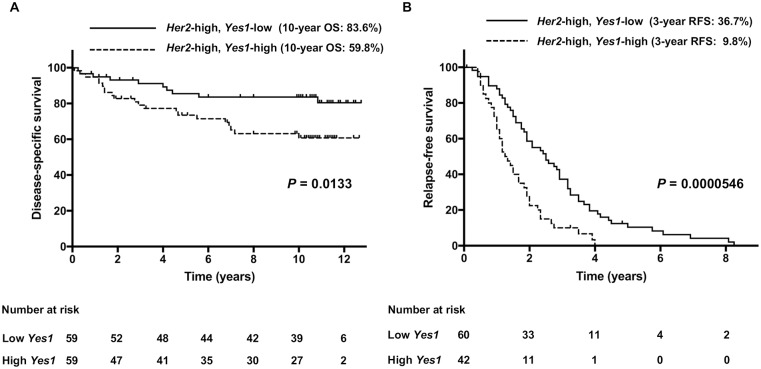
Effect of the *Yes1* mRNA expression on prognosis in HER2-positive breast cancer patients. (A) In *TP53* cohort (GSE3494) with higher expression of *HER2*, disease-specific survival (DSS) of the cases with higher *Yes1* mRNA expression was significantly shorter than that of the cases with lower expression of *Yes1* (P = 0.0133). (B) In metastasis cohort (GSE12276) with higher expression of *HER2*, relapse free survival (RFS) of the cases with higher expression of *Yes1* was significantly shorter than that of the cases with lower expression of *Yes1* (P = 0.0000546).

We further investigated whether overexpression of Yes1 had any impact on clinicopathological characteristics of breast cancers. To this end, we examined the associations of *Yes1* mRNA expression with prognosis of breast cancer patients using two independent datasets, such as 236 breast cancer patients with/without *TP53* mutation (*TP53* cohort, GSE3494) and 204 breast cancer patients with distant metastasis (metastasis cohort, GSE12276). Patients could be divided into low and high expression groups of *HER2* mRNA, as well as *Yes1* mRNA, based on the median expression value of all the patients within the dataset, respectively. In *TP53* cohort with higher expression of *HER2* (n = 118), disease-specific survival (DSS) of the cases with higher expression of *Yes1* was significantly shorter than that of the cases with lower expression of *Yes1* (P = 0.0133, Fig 6A). Ten-year DSS rate for higher and lower expression of *Yes1* was 59.8% and 83.6%, respectively. In addition, relapse free survival (RFS) of the cases with higher expression of *Yes1* was significantly shorter than that of the cases with lower expression of *Yes1* (P = 0.0000546, Fig 6B) in metastasis cohort with higher expression of *HER2* (n = 102). Three-year RFS rate for higher and lower expression of *Yes1* was 9.8% and 36.7%, respectively. These findings suggest the cooperative expression of Yes1 and HER2 associates with poor prognosis in breast cancer patients.

## Discussion

Our findings demonstrated that copy number gain of *Yes1*, which encodes Src family protein Yes1, was a mechanism of the acquired resistance to trastuzumab and lapatinib. As lapatinib is used in the situation of the acquired resistance to trastuzumab in HER2-positive breast cancers clinically, we established a trastuzumab/lapatinib-dual resistant cell line (BT-474-RL2) from trastuzumab-resistant cell line (BT-474-R). Of interest, *Yes1* copy number was more increased in BT-474-RL2 compared to BT-474-R, resulting in further increased mRNA and protein expression of Yes1 in BT-474-RL2. Src family proteins are non-receptor tyrosine kinases, and are reported to accelerate cell proliferation by the reciprocal activation with RTKs such as HER2, and activating PI3K/Akt and MAPK pathways [16, 27]. Src is considered as an oncogene, as it is reported to be dysregulated in breast and colorectal cancers [28, 29]. It is reported that the activation of Src family proteins is associated with the acquired resistance to trastuzumab in gastric cancer [30]. Src family proteins are also reported to be associated with the resistance to lapatinib [31]. Src family proteins include c-Src Yes, Fyn, Lyn, Fgr, Blk, Hck, Lck, and Frk [32]. Thus, we further analyzed which Src protein was related with the mechanism of the resistance to trastuzumab and lapatinib, resulting in the clarification of aberrant expression of *Yes1*. Knockdown of *Yes1* resulted in the recovery of the sensitivity to trastuzumab and lapatinib, indicating that Yes1 was the key molecule to overcome the resistance to trastuzumab and lapatinib.

There are many works elucidating the resistance mechanisms of trastuzumab, and several mechanisms were reported. Even in the case that the same cell line, BT-474, is used, different mechanisms have been reported. For example, we had found that nuclear factor-kappa B (NF-kappaB) was constitutively activated in the BT-474-R cells [23]. In the process of acquiring the resistance, several different molecular alterations may emerge depending on the culture condition *in vitro* and microenviromental conditions *in vivo*. We previously reported that drug concentration in the process of establishing the resistant cells to gefitinib contributed to the different causative alterations in the acquired resistant clones derived from the same parental cancer cell lines [33]. In the current study, our experimental results documented that Src family Yes1 was responsible to the resistance. Because Src has already been reported as the resistance mechanism of trastuzumab [16, 17], we consider that Src signaling plays a pivotal role in the acquired resistance to trastuzumab.

We focused on dasatinib, which is known to be a Src inhibitor and is used for targeting BCL-ABL fusion protein in chronic myelocytic leukemia and for targeting Philadelphia chromosome in acute lymphatic leukemia [34]. Dasatinib also targets Yes1 [35]. One of the strategy to conquer the resistance to a certain drug targeting driver alteration is the combination treatment of the drug targeting driver alteration and the one targeting the mechanism of the resistance to driver alteration. For example, *MET* amplification can be detected after the treatment of the first generation of EGFR-tyrosine kinase inhibitor (EGFR-TKI), gefitinib or erlotinib, in *EGFR*-mutant lung adenocarcinomas, and the combination of EGFR-TKI and MET inhibitor is demonstrated to be an effective treatment [36]. In the current study, dasatinib plus trastuzumab or lapatinib showed additive anti-tumor effect to BT-474-R and BT-474-RL2, although dasatinib alone had an enough anti-tumor effect to the cells resistant to trastuzumab or lapatinib. Acquired resistance of cancer cells to a drug targeting the driver alteration can be mediated by a switch to the dependency on a certain molecule [37, 38]. In the acquisition of the resistance to trastuzumab/lapatinib, there is a possibility that a switch to Yes1 was induced to breast cancer cells depending on HER2 for proliferation, as dasatinib alone had an enough power to treat the resistant cells. Regarding the discrepancy of the results between Fig 4A and 4B, we consider that the difference of the drug exposure time may influence the results. Fig 4A shows the result of cell viability assay, in which the cells were exposed to drugs for 72 hours. On the other hand, the cells were exposed to the drugs for 14 days in clonogenic assay shown in Fig 4B. Additionally, these two types of assays measure different properties of cancer cells. Cell viability assay using MTS are set up at higher cell densities in the plate and cell proliferation in the presence of the range of drug concentration is measured. On the other hand, clonogenic assay measures clonogenicity of cancer cells, which involves different signaling pathways. As for another reason why dasatinib alone showed the inhibitory effect of cell proliferation to the resistant cell lines compared to the Yes1-specific inhibition by siRNA, we consider that dasatinib is a multi-kinase inhibitor, and thus dasatinib inhibits not only Src family member proteins but also BCR/ABL, c-Kit, EPHA2 receptors, and PDGF beta receptors, which might partially contribute on cell survival, resulting in the inhibitory effect. Although Src inhibitor saracatinib was reported to overcome the resistance mechanism [16, 30], saracatinib is not applied to clinical setting at the present moment. On the other hand, dasatinib has already been used in clinical setting and it can be applied to the treatment to conquer the resistance to trastuzumab and lapatinib.

We also conducted *in vivo* analysis after injecting BT-474, BT-474-R to severe combined immunodeficiency (SCID) mice. However, BT-474-R did not show enough resistance to trastuzumab *in vivo* (S2 Fig). This is considered due to antibody-dependent-cellular-cytotoxicity (ADCC) in SCID mice [39]. As for BT-474-RL2, tumor formation in SCID mice was not enough to perform *in vivo* analysis.

To elucidate the impact of Yes1 expression on the prognosis in HER2-positive breast cancer patients, we performed bioinformatics analysis using two different cohorts. Higher *Yes1* expression was correlated with worse prognosis in HER2-positive breast cancer patients. Although the poor prognostic factor is not strictly equivalent to the resistance mechanism to trastuzumab, the results from clinical data support that Yes1 has a pivotal role in HER2-positive breast cancer.

In resistant cell lines, the phosphorylation of HER2 and Akt was upregulated in addition to Yes1, whereas phospho-MAPK was downregulated, implying that MAPK signaling might be inhibited by the negative feedback due to the activation of PI3K/Akt pathway. To understand this result, a further study is necessary.

In conclusion, we found that Yes1 is a key molecule for the resistance mechanism of trastuzumab and lapatinib in a certain population of HER2-positive breast cancers. Further investigation is warranted to clarify the mechanism of the acquired resistance to trastuzumab and lapatinib and for the therapeutic strategy to overcome the resistance.

## Supporting information

S1 Fig *Blk*, *Fgr* and *Hck* are highly expressed in lymphoma cell lines. *Blk* and *Fgr* were expressed in Burkitt’s lymphoma cell line Daudi, whereas *Hck* was expressed in histiocytic lymphoma cell line U937. However, these genes were not expressed in BT-474, BT-474-R or BT-474-RL2.
(TIF)

S2 Fig Growth of BT-474 and BT-474-R in SCID mice. The animals were treated with vehicle or 2 mg/kg trastuzumab once per week (arrows). Data are shown as means ± standard deviation (SD) (n = 4).
(TIF)

S3 Fig Original data of western blot analysis in Figs 2–5 and RT-PCR in S1 Fig. Original data of western blot analysis and RT-PCR is provided.
(PDF)

S1 Method Methods for “mRNA expression of *Blk*, *Fgr* and *Hck*” and “Animal xenograft mouse model”. Detailed methods are described.
(DOCX)
