# Correction: Yes1 signaling mediates the resistance to Trastuzumab/Lap atinib in breast cancer

**DOI:** 10.1371/journal.pone.0174493

**Published:** 2017-03-16

**Authors:** The PLOS ONE Staff

In the article title, “Lap atinib” should be displayed as “Lapatinib.” The correct title is: Yes1 signaling mediates the resistance to Trastuzumab/Lapatinib in breast cancer. The correct citation is: Takeda T, Yamamoto H, Kanzaki H, Suzawa K, Yoshioka T, Tomida S, et al. (2017) Yes1 signaling mediates the resistance to Trastuzumab/Lapatinib in breast cancer. PLoS ONE 12(2): e0171356. doi:10.1371/journal.pone.0171356. The publisher apologizes for the error.

## References

[1] Takeda T, Yamamoto H, Kanzaki H, Suzawa K, Yoshioka T, Tomida S, et al (2017) Yes1 signaling mediates the resistance to Trastuzumab/Lap atinib in breast cancer. PLoS ONE 12(2): e0171356 doi:10.1371/journal.pone.0171356 28158234 10.1371/journal.pone.0171356 PMC5291431

