# Supplementary figures and images for: Yes1 signaling mediates the resistance to Trastuzumab/Lap atinib in breast cancer
Source: PLoS One. 2017 Feb 3;12(2):e0171356. doi: 10.1371/journal.pone.0171356 (PMC5291431; doi:10.1371/journal.pone.0171356)

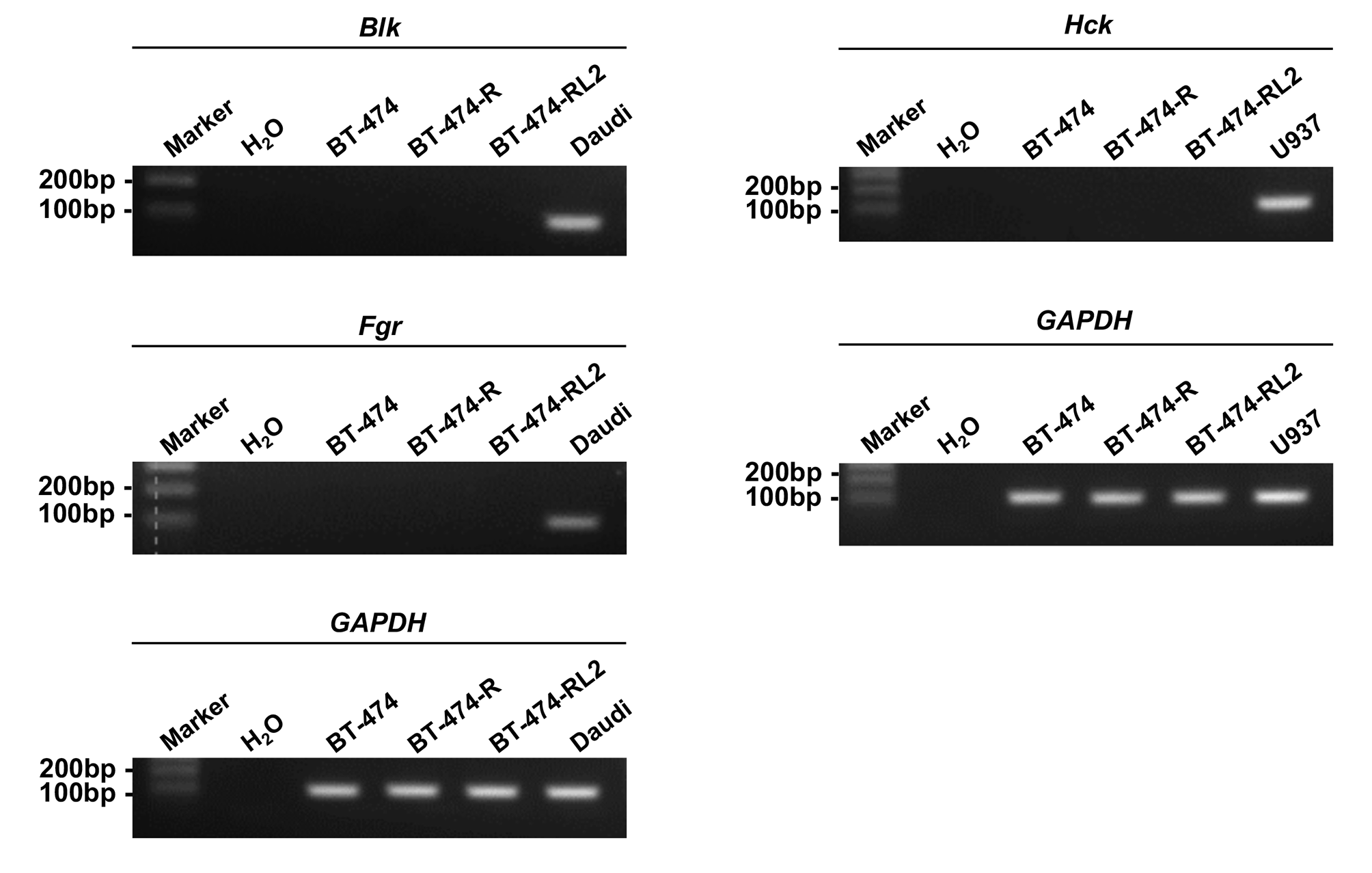

Supplement: S1 Fig — Blk and Fgr were expressed in Burkitt’s lymphoma cell line Daudi, whereas Hck was expressed in histiocytic lymphoma cell line U937. However, these genes were not expressed in BT-474, BT-474-R or BT-474-RL2. (TIF) [file pone.0171356.s001.tif]

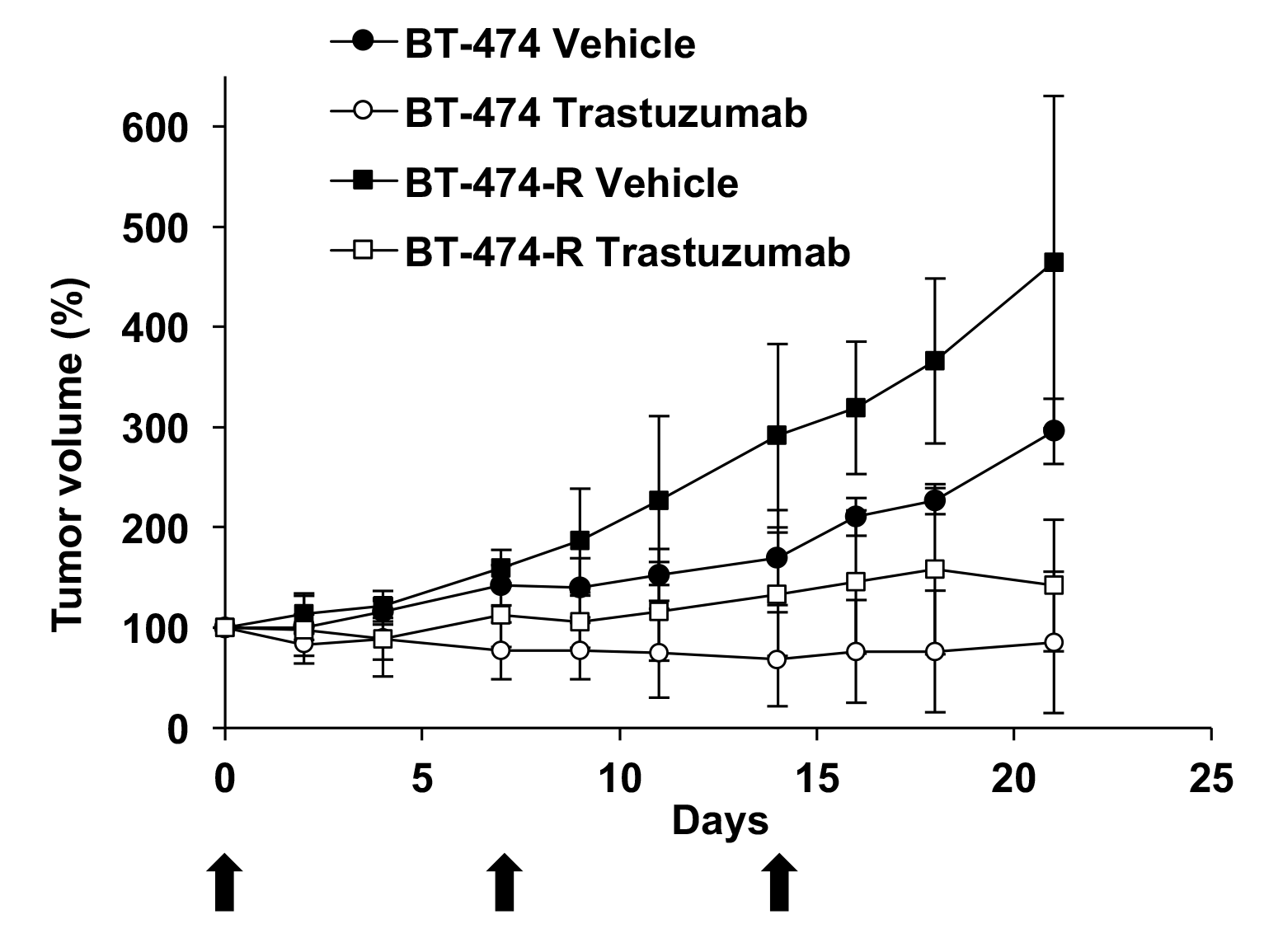

Supplement: S2 Fig — The animals were treated with vehicle or 2 mg/kg trastuzumab once per week (arrows). Data are shown as means ± standard deviation (SD) (n = 4). (TIF) [file pone.0171356.s002.tif]

Fig 2B

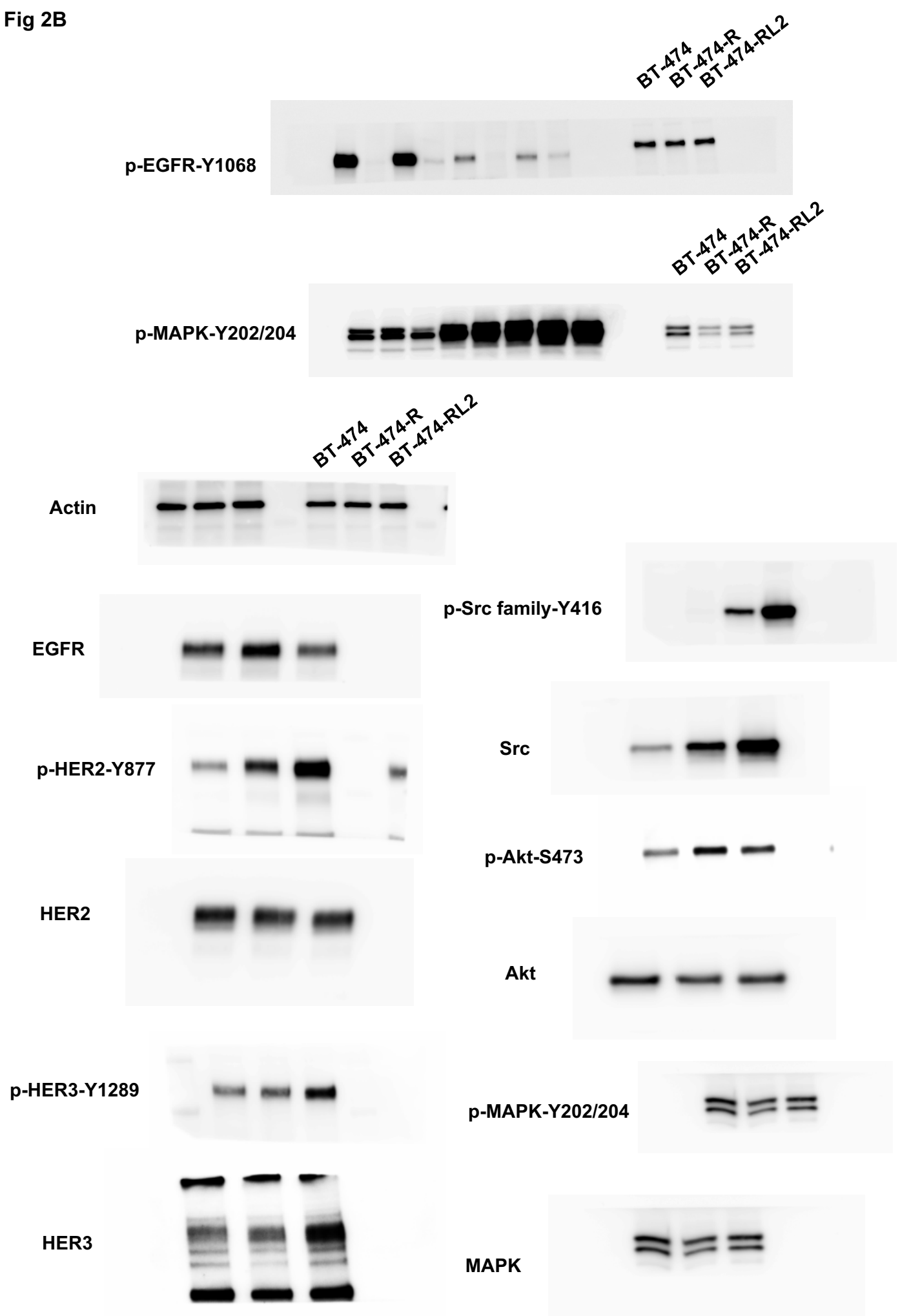

Fig 3B

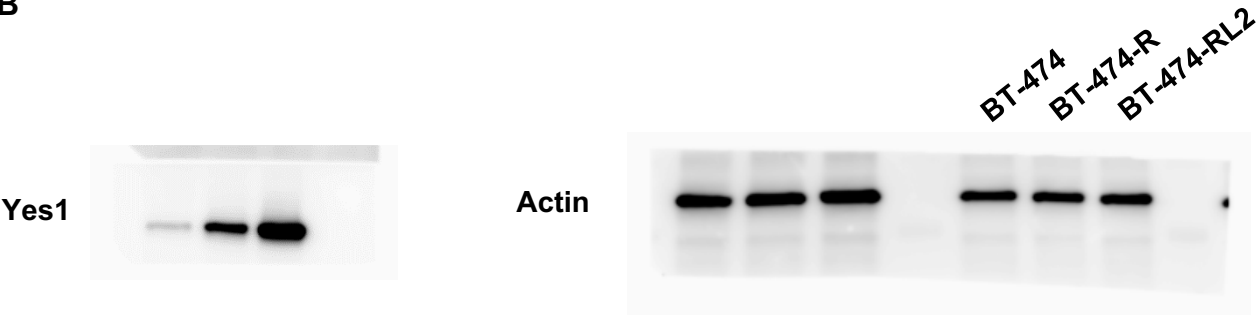

Fig 3D

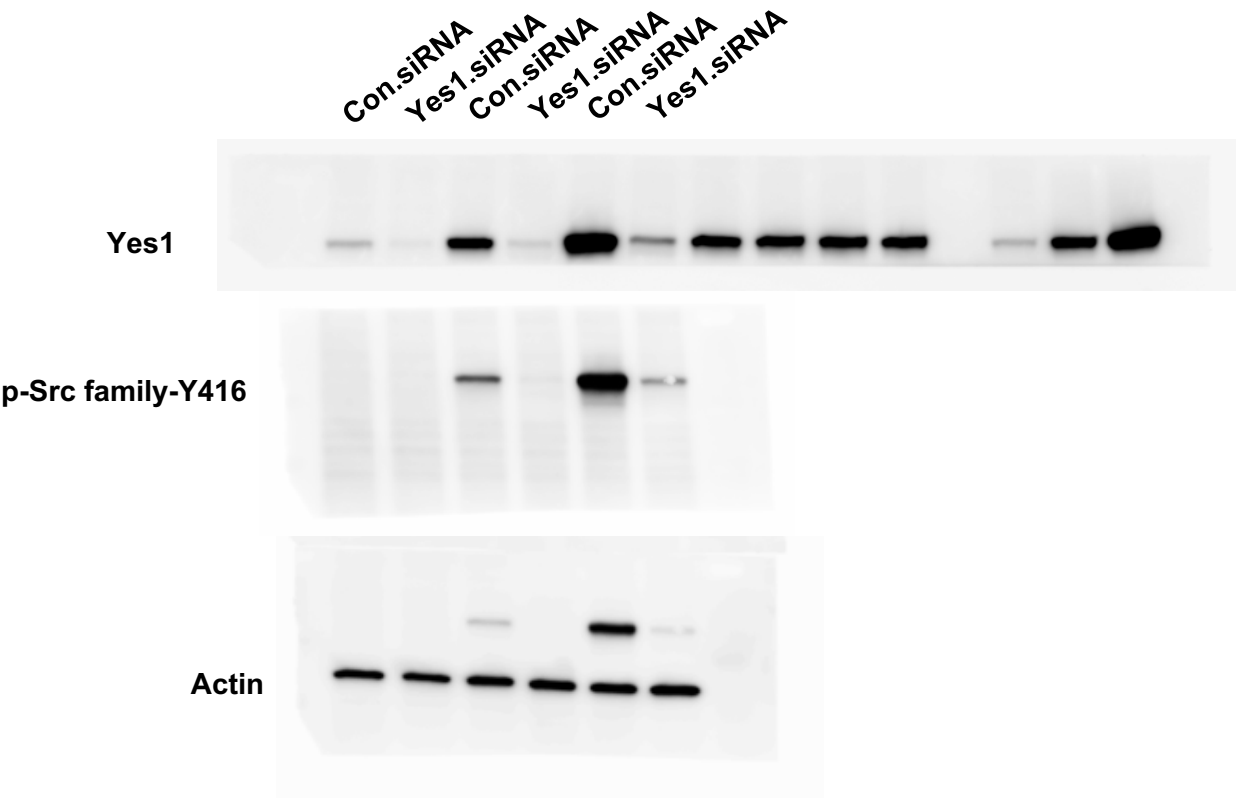

**Fig 4D**

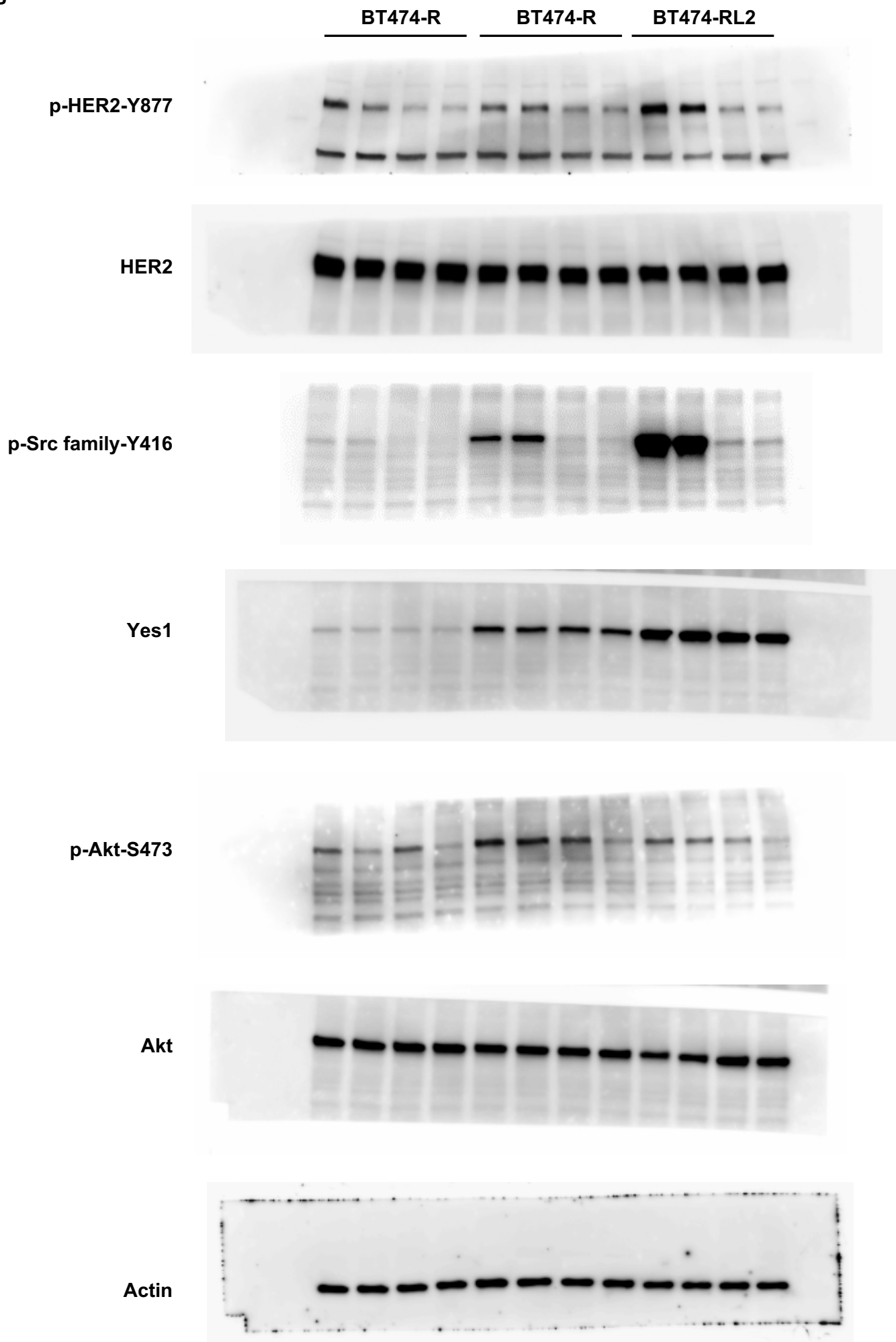

Fig 5B

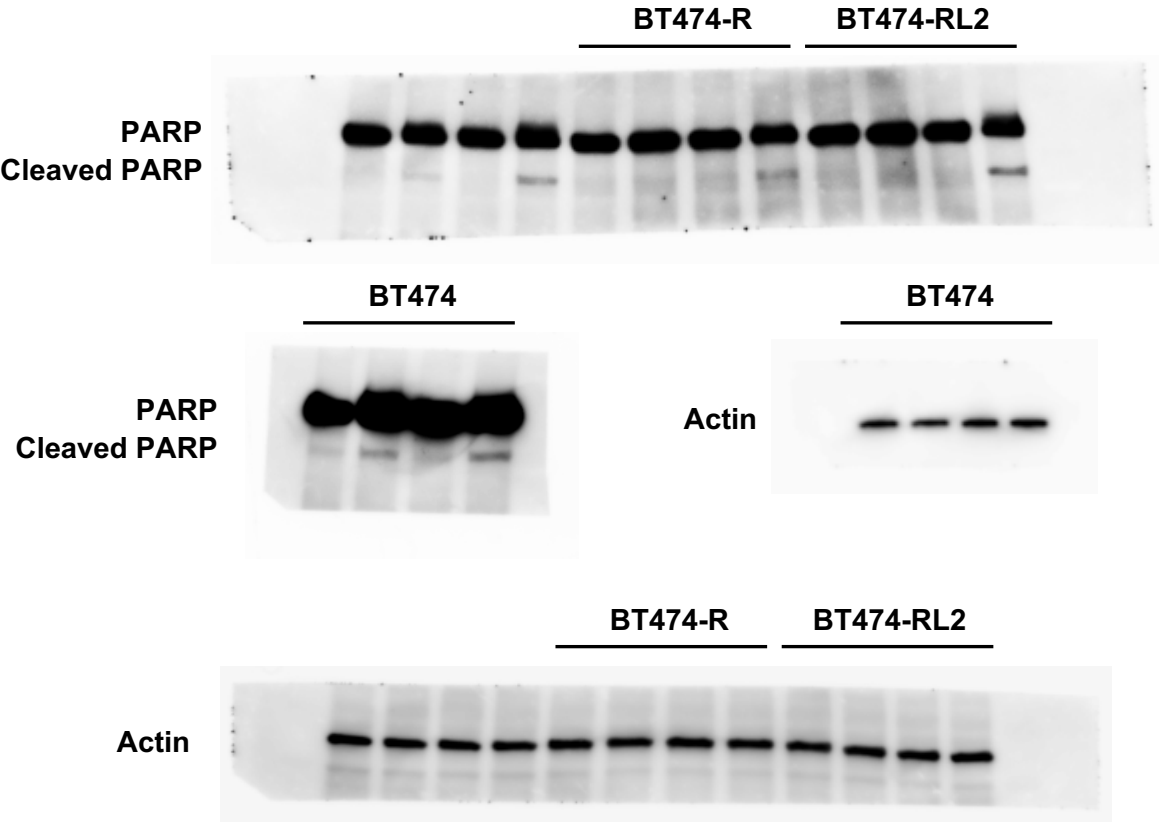

S1 Fig

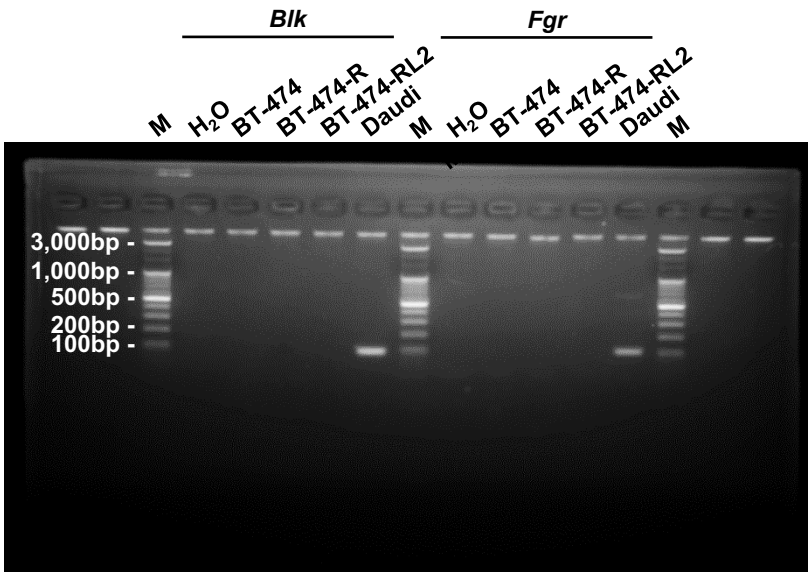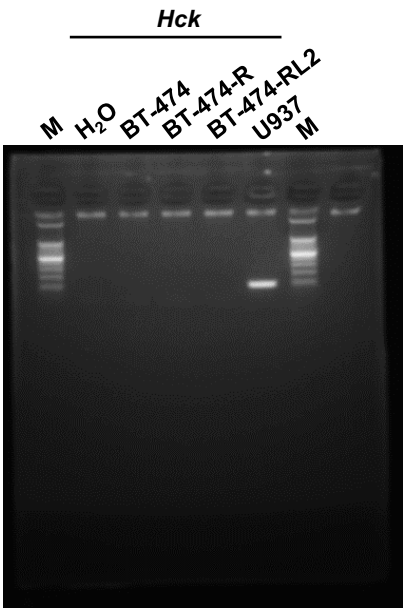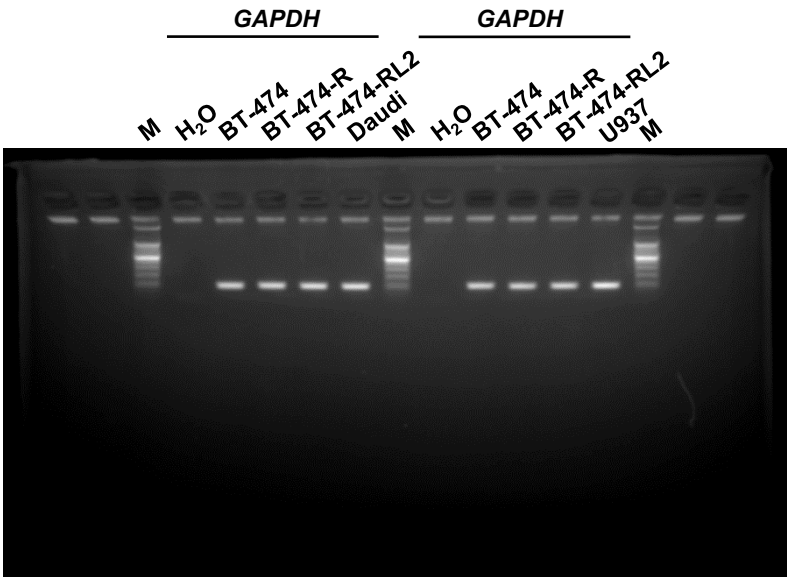

Supplement: S3 Fig — Original data of western blot analysis and RT-PCR is provided. (PDF) [file pone.0171356.s003.pdf]
