# Supplementary material for: Yes1 signaling mediates the resistance to Trastuzumab/Lap atinib in breast cancer
Source: PLoS One. 2017 Feb 3;12(2):e0171356. doi: 10.1371/journal.pone.0171356 (PMC5291431; doi:10.1371/journal.pone.0171356)
Supplement: S1 Method — Detailed methods are described. (DOCX) [file pone.0171356.s004.docx]

**Takeda et al.**

**Yes1 Signaling Mediates the Resistance to Trastuzumab/Lapatinib in Breast cancer**

**S1 Method**

**mRNA expression of *Blk*, *Fgr* and *Hck***

Burkitt’s lymphoma cell line Daudi (catalog number: JCRB9071) and histiocytic lymphoma cell line U937 (catalog number: JCRB9021) were purchased from Japanese Collection of Research Bioresources Cell Bank (Tokyo, Japan). The cells were maintained in RPMI with 20% fetal bovine serum (Daudi) or 10% fetal bovine serum (U937) at 5% CO_2_ under 37°C. Total RNA was isolated and reverse transcribed as described in the Materials and Methods of main text. The reverse transcription PCR was performed on Veriti™ 96-Well Thermal Cycler (Thermo Fisher Scientific) using HotStarTaq *Plus* DNA Polymerase (Qiagen). PCR products were electrophoresed in 2% agarose gels with ethidium bromide and detected using ImageQuant LAS 4000 (General Electric Company). 100bp DNA Ladder (FUKAEKASEI Co., Ltd, Kobe, Japan) was used for marker.

**Animal xenograft mouse model**

Six-week-old female severe combined immunodeficiency (SCID) mice were obtained from Charles River Laboratories (Yokohama, Japan). All mice were provided with sterilized food and water, and housed in a barrier facility under a 12:12-h light:dark cycle. Each cell line (5 × 10^6^) was suspended with 200 μL of DMEM:Matrigel Basement Membrane Matrix (Corning Inc., Corning, NY, USA) mixture (1:1 ratio) and subcutaneously injected into the backs of the mice. Tumors were measured using digital calipers, and each tumor volume was calculated using the formula: volume= 1/2 × [(shortest diameter)^2^× (the longest diameter)]. When the tumors exceeded approximately 100 mm^3^, mice were randomly allocated to two groups that received either vehicle or 2 mg/kg trastuzumab. Vehicle and trastuzumab were given by intravenous injection, once per week. Tumor volume was measured three times a week. The protocol was approved by the Animal Care and Use Committee, Okayama University (Permit Number: OKU-2014635).
